# The Role of CT Arthrography in Shoulder Instability

**DOI:** 10.5334/jbr-btr.1208

**Published:** 2016-11-19

**Authors:** Reto Sutter

**Affiliations:** 1 Balgrist University Hospital, Zurich, Switzerland

## Abstract

Computerized Tomography (CT) and CT arthrography are useful tools in evaluating both the osseous structures and the soft tissues in patients with shoulder instability. A variety of osseous injuries can be accurately depicted, such as Hill-Sachs lesions, glenoid rim fractures, or glenoid bone loss. CT arthrography further allows precise evaluations of labral and chondral defects. Further, CT is a simple modality for performing anatomical measurements in the shoulder such as glenoid version or for the assessment of osseous deficiencies of the posterior glenoid. Finally, CT is also be beneficial for assessing patients in the postoperative situation.

The shoulder is the most commonly dislocated joint in adults. The majority of shoulder dislocations are caused by trauma and are usually unidirectional, with the anteroinferior shoulder instability being the most common [1]. Much less common are posterior and multidirectional instabilities. Computerized Tomography (CT) and CT arthrography are useful tools in evaluating both the osseous structures and the soft tissues in patients with shoulder instability. The injection for CT arthrography can be performed under fluoroscopy guidance, under sonography, or even directly on the CT examination table with a low-dose CT protocol for the injection itself, followed by the standard diagnostic CT [2].

In patients with classic anteroinferior shoulder instability commonly a posterosuperior osseous impression of the humeral head can be found, the so called Hill-Sachs defect (Figure 1). With CT this can be easily diagnosed and quantified. In posterior shoulder instability CT may show an inversed Hill-Sachs defect [3]. CT is the most accurate modality to assess the glenoid bone; whereas the amount of osseous defects and glenoid bone loss at the anterior part of the glenoid may be underestimated with use of magnetic resonance imaging (MRI) in patients with anteroinferior shoulder instability, CT allows a precise visualization of this part of the glenoid, both in cases with acute glenoid fracture and in cases with chronic instability. In cases with only minor glenoid bone loss a labral repair and capsular surgery may be performed, while in cases with substantial glenoid bone loss, usually an osseous corrective surgery is preferred [4].

**Figure 1:**
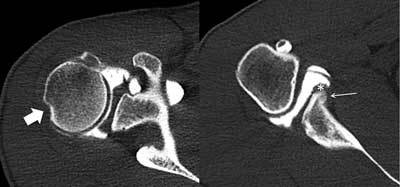
CT arthrography of the right shoulder in a 24-year-old male patient with anteroinferior shoulder instability demonstrating a Hill-Sachs defect (broad arrow on left image) posterosuperior on the humeral head on a transverse image. More caudally (right image) a scarred and medialized labrum can be found (asterisk) adjacent to a healed anterior glenoid rim fracture with residual callus (thin arrow).

With its inherent high spatial resolution, CT arthrography is useful for the assessment of the biceps anchor, the glenoid labrum and capsule-labrum complex and also for a precise evaluation of articular cartilage defects [5,6]. CT is also often used to evaluate the glenoid version, and to assess atrophy and fatty infiltration of the rotator cuff muscle. For the latter, sagittal CT reconstructions of the shoulder joint should extend medially to cover the complete rotator cuff muscles. Abnormal glenoid version has been linked to an increased risk of chronic anterior or posterior shoulder instability with angles that deviated more than 15° the neutral position [7]. However, an increased retroversion of the glenoid is not associated with a higher risk of first-time posterior shoulder dislocation [8].

For patients with suspected posterior shoulder instability, CT is also useful in quantifying the amount of osseous deficiency of the posterior glenoid. Ninety-three percent of patients with recurrent atraumatic posterior shoulder instability show a bony deficiency of the posterior glenoid, compared to 60–73% of patients without posterior instability [9].

Finally, CT is beneficial for assessing patients in the postoperative situation, e.g. after a Latarjet procedure (where the distal part of the coracoid process is transferred to the anterior portion of the glenoid in order to prevent re-dislocation of the shoulder joint) [10]. CT allows to accurately assess the position of the osseous block and to detect a possible non-union of the osseous block. In patients with a suspected dislocation of anchors after rotator cuff repair CT allows the identification and localization of the anchors and surgical wires.
